# The At Risk Child Clinic (ARCC): 3 Years of Health Activities in Support of the Most Vulnerable Children in Beira, Mozambique

**DOI:** 10.3390/ijerph15071350

**Published:** 2018-06-27

**Authors:** Claudia Marotta, Francesco Di Gennaro, Damiano Pizzol, Geoffrey Madeira, Laura Monno, Annalisa Saracino, Giovanni Putoto, Alessandra Casuccio, Walter Mazzucco

**Affiliations:** 1 Department of Science for Health Promotion and Mother to Child Care “G. D'Alessandro”, University of Palermo, Via del Vespro, Palermo 90217, Italy; claudia.marotta@unipa.it (C.M.); alessandra.casuccio@unipa.it (A.C.); walter.mazzucco@unipa.it (W.M.); 2 Department of Infectious Diseases, University of Bari “Aldo Moro”, Bari 70124, Italy; laura.monno@uniba.it (L.M.); annalisa.saracino@gmail.com (A.S.); 3 Research Unit, Doctors with Africa CUAMM, Beira 1363, Mozambique; d.pizzol@cuamm.org; 4 Instituto Nacional de Saúde, Maputo 116 Mozambique; geogirassol@gmail.com; 5 Research Section, Doctors with Africa CUAMM, Padova 35128, Italy; g.putoto@cuamm.org

**Keywords:** Mozambique, children at risk, HIV-exposed infants (HEI), Mother and Child Health (MCH) services, children’s health

## Abstract

The concept of “children at risk” changes worldwide according to each specific context. Africa has a large burden of overall risk factors related to childhood health and development, most of which are of an infective or social origin. The aim of this study was to report and analyze the volumes of activities of fifteen At Risk Child Clinics (ARCCs) within the Beira District (Mozambique) over a 3 year-period in order to define the health profile of children accessing such health services. We retrospectively analyzed the data from all of the children accessing one of the 15 Beira ARCCs from January 2015 to December 2017. From this, 17,657 first consultations were registered. The motivations for accessing the services were in order of relevance: HIV exposure (n. 12,300; 69.7%), other risk conditions (n. 2542; 14.4%), Moderate Acute Malnutrition (MAM) (n. 1664; 9.4%), Severe Acute Malnutrition (SAM) (n. 772; 4.4%), and TB exposure (n. 542; 3.1%). During the first consultations, 16,865 children were screened for HIV (95.5%), and 7.89% tested HIV-positive. In our three years of experience, HIV exposure was the main indication for children to access the ARCCs in Mozambique. ARCCs could represent a strategic point to better understand health demands and to monitor the quality of care provided to this vulnerable population group, however significant effort is needed to improve the quality of the data collection.

## 1. Introduction

The concept of “children at risk” is dynamic worldwide, so the health risk profile of children is changing according to each specific geographic and social context. In the African continent, the greatest risks to children’s health and development are mainly of infective and social origin, particularly exposure to Human Immunodeficiency Virus (HIV), malaria, TB, and malnutrition [1]. It is no coincidence that the scope for child development improvement at the regional level can be identified within three domains: nutrition, environment, and mother-child interaction [1].

In low income countries, despite the increasing investments in effective prevention strategies, HIV mother to child transmission (MTCT) remains a significant problem, especially in sub-Saharan Africa [2] where AIDS is still among the top causes of under-five morbidity and mortality [3]. In particular, in 2015, Mozambique was estimated to contain about 94,000 HIV-positive pregnant women, approximately 15% of whom transmitted HIV to their newborn infants, resulting in nearly 14,000 new pediatric HIV infections [2].

Again, tuberculosis (TB) in endemic settings is an under recognized, but potentially significant cause of morbidity and mortality in children [4,5]. In Mozambique, pediatric TB accounted for 7% of all new cases reported in 2012 [6]. Further, with 58% of all reported TB cases being HIV-positive, Mozambique also has one of the highest TB/HIV co-infection rates [7]. However, national data reported a decreasing trend in the under-5 mortality rate, with 71.3 deaths per 1000 live births in 2016, and among the top causes of post-neonatal death being Pneumonia, Malaria, and AIDS [8].

The impact of undernutrition on children’s morbidity and mortality in Sub–Saharan Africa is also well known [9,10,11,12,13]. In Mozambique, according to the latest data from UNICEF, 43% of children suffer from moderate or severe stunting [8], and since children constitute 52% of the population in the country [14], it constitutes a very big health issue for the country.

If, on one hand, the deep impact of health determinants such as poverty and social disparities of child health are well known [15,16], then great attention should be paid to the emerging risks, such as climate change and environmental pollution, whose specific role on child health and development has only been better investigated in recent years [15,17,18].

In this epidemiological framework, with specific regard to health services, while consistent evidence is available for antenatal care [2,19,20], very little fragmented information on the postnatal care activities has been provided—especially for vulnerable children and their mothers.

The aim of this study was to report and analyze the volumes of activities of fifteen At Risk Child Clinics (ARCCs) within the Beira District over a 3 year-period in order to define the health profile of the children accessing these specific types of health services.

## 2. Materials and Methods 

### 2.1. Study Setting

The Beira District is one of the 13 districts of the Sofala Province, laying on the eastern coast of Mozambique, and it is the third largest district in the country. The health system of the Sofala Province is articulated in 146 health facilities (1 per every 12,000 inhabitants) [21] and it is organized into four basic levels of care, including (1) one quaternary-level hospital in Beira, (2) four secondary-level rural hospitals, (3) 114 urban and rural health centers, including Maternal and Child Health Services specific to mothers and children, which are managed by the Ministry of Health (MoH), and (4) 27 health posts [22].

The ARCCs are configured as specific clinics within the Maternal and Child Health Services, and since 2012, most of the 15 ARCCs of the Beira district are supported by Doctors with Africa CUAMM.

Each ARCC provides free out-patient consultations dedicated to new-borns and children under five with specific health risks, such as HIV exposed infants (HEI), preterm, malnourished, TB exposed, referred to Maternal and Child Health Services from maternity, health posts, neonatology, or directly accessing the MCH services. Children that are presented in centers are taken charge of by clinical officers and nurses who provide care in an out-patient setting. The ones requiring more complex care and/or admission are addressed to the Beira referral hospital or to chronic disease out-patient services, except for HEI. In fact, since 2013, after a positive screening test with a PCR or Rapid test, a confirmation test with Western Blot [23] has been performed and, if positive again, the HEI remains in charge of ARCCS until 5 years old, together with his mother, in order to guarantee a better continuum of care [24].

### 2.2. Study Population and Period

We retrospectively analyzed all children accessing one of the 15 Beira ARCCs for a first consultation over a 3-year period, from January 2015 to December 2017.

### 2.3. Data Collection and Analysis

Routine service data were accessed. Data collection in Health Centers (HC) goes through several processes, from registration to assignment to the higher levels (Provincial and National). Firstly, data is recorded in each health section of HC during the consultation using a national format in a daily logbook. Health professionals have to ensure consistency and completeness (filling in all fields) of the registration in order to obtain high quality data. The daily logbook is filled exclusively by the health care professional who delivers the service. At the end of the activities, the daily summary is elaborated on. In this case, the person in charge of this activity verifies the agreement of the data. After the last day of the period under analysis (following the statistical calendar), a monthly summary is made, aggregating the daily summaries corresponding to the period in question. The direction of the HC is responsible for issuing and approving the summaries. Thus, the data analysis is done at the HC level and, subsequently, is reported to the district level and is then sent to higher levels.

We accessed the data at the district level, obtaining aggregate information on a number of consultations at each ARCCs. Information about the motives of the first consultation, the type and the timing of the first HIV test that was administered and it’s result, and the type and duration of breastfeeding were also extracted. Motivations were grouped into 5 categories: TB exposure, Moderate Acute Malnutrition (MAM), Severe Acute Malnutrition (SAM), HIV exposure, and other risk conditions (e.g., malaria, preterm). 

Moderate Acute Malnutrition and Severe Acute Malnutrition were defined according to the z-scores of weight-for-height [25,26].

A database was created on Microsoft Excel software and was analyzed using the STATA 13.0 statistics software. The frequencies for the categorical variables were calculated as descriptive statistics.

Data use for publication was approved by the District Health Authority in Beira, the Health District Direction (protocol reference: 293 /15), Mozambique.

## 3. Results

During the period between 2015 and 2017, an overall number of 17,657 first consultations were registered at the 15 different ARCCs serving the Beira district, representing 50.5% [27] of the 34,968 estimated new-borns in the study period.

Table 1 reports the first consultations per each one of the 15 ARCCs in the Beira District over the 3-year study period, ranging from a minimum of 92 first consultations for Marrocanhe to a maximum of 3105 for M-Nhaconjo.

The motivations for accessing the services were in order of relevance, (a) HIV exposure (n.12,300; 69.7 %), (b) other risk conditions (n. 2542; 14.4%), (c) MAM (n. 1664; 9.4%), (d) SAM (n. 772; 4.4%), and (e) TB exposure (n. 542; 3.1%) (Table 1). HIV exposure was the most frequent motive that was reported, with the exception of Mataduro where 54.9% (n. 62) of the children were referred to a MAM (Table 1).

During the first consultations that were done at the ARCCs of the Beira District, over the period between 2015 and 2017, 16,865 children were screened for HIV (95.5%) overall, regardless of their exposure status (Table 2).

Polymerase chain reaction (PCR) examination was tested in 8437 (49.5%) children before the 8th week and in 2216 (31.1%) between the 8th week and the 9th month. The remaining 6302 (37.4%) children were screened with a rapid test that was executed between the 9th and the 18th month (Table 2).

Of the 16,865 children who were screened for HIV, independent from their HIV exposure status, 1330 (7.89%) documented a sero-positivity. Overall, the prevalence of HIV sero-positivity was 13.3% (n. 295) among the patients who were tested with PCR between the 8th week and the 9th month, 8.9% (n. 561) among the patients who were tested with the Rapid Test between the 9th and 18th month, and 5.6% (n. 474) among the ones who were tested with PCR before the 8th week of life (Table 2). The percentages of the specific timing, type, and results of the first HIV test that was made at each ARCC are shown in Table 2.

Data on breastfeeding type was available for 10,100 children (82.1%) on 12,300 HIV-Exposed infants who were accessing the At Risk Children Clinics (ARCCs) over the study period. Eighty-three percent (n. 8405) of them were currently and exclusively breastfed by their mothers, while 11.3% were fed with formula, and 5.5% were fed with mixed breastfeeding. A similar distribution of breastfeeding type is reported by ARCC (Table 3).

## 4. Discussion

We reported data from three years of activity that was conducted at ARCCs in the Beira District, Mozambique, using the routinely collected data in order to provide an indirect picture of the health status of children under five who were accessing the local health facilities.

Over the three years of the study, HIV exposure represented the main reason for access to ARCC for consultation, as 7.89% of all of the children who visited were HIV-positive.

Of interest is that 83% of the HIV-Exposed Infants accessing the ARCCs over the study period were currently and exclusively breastfed by their mothers, while 10% were fed with formula, and 5% with mixed feeding. Since the HIV status of the mothers and the age of the children were unknown, it is not possible to draw consistent conclusions on adherence to what is recommended by WHO for HEI breastfeeding [28], however even from our inaccurate and rough data, it seems to be far from what was expected. Actions should be taken to improve the exclusive breastfeeding rate in this category, whereas mixed feeding increases the risk of HIV transmission [29,30] and poses the same risks of contamination and diarrhea as artificial feeding, which ultimately can affect infant survival.

The main limitation in the interpretation of the information that has been reported is related to the nature of the study. In fact, since we performed a retrospective study using only health data that was routinely collected, we were not able to access complete information about many aspects that were of interest. In particular, it could have been important to understand what is categorized under “other conditions”, so as to extrapolate the Malaria cases, since Malaria is a leading cause of death among children in Mozambique and it causes important sequels in the surviving ones, including neuro-cognitive damage [31]. Also, knowing the burden of the patients who access care for more than one health problem could have been informative since most of the unfavorable conditions came together: a child with HIV has a higher risk of undernutrition and presents a higher risk of disease progression and mortality [9,10,11,12].

Furthermore, infants and young children (<3 years) and those with immunodeficiency caused by HIV or severe malnutrition are at the highest risk of developing TB [6], which is demonstrated to be the main cause of death among under-fives, accounting for 26% of AIDS-related deaths [7]. Moreover, to date, we are not able to estimate the number of children who are not born in health facilities that are supposed to be at a higher risk, which may be the ones who are not accessing the ARCC.

Despite the aforementioned limitations, ARCCs could represent a strategic point to better understand health demand and to monitor the quality of care that is provided to this vulnerable population group, however important efforts are needed to improve the quality of the data collection.

## 5. Conclusions

Children at risk are among the most vulnerable population group, which means that constant attention should be paid to their health needs, particularly in developing countries. Therefore, follow-up data collected at ARCC on children at risk and their clinical outcomes should be analyzed in order to monitor the quality of the care provided and to implement strategies for improvement. 

To date, ARCCs represent a strategic source of information to address health policies and health programs [32].

## Figures and Tables

**Table 1:** First consultations, by motivation and overall, at ARCCs of the Beira District, 2015–2017.

ARCCs of the Beira District	TB Exposure n. (%)	Moderate Acute Malnutrition n. (%)	Severe Acute Malnutrition n. (%)	HIV Exposure n. (%)	Other Risk Conditions n. (%)	Total First Consultations n. (%)
Chamba	9 (1.4)	54 (8.3)	18 (2.8)	466 (71.8)	102 (15.7)	649 (100)
Chingussura	73 (3.2)	94 (4.1)	68 (2.9)	1822 (78.7)	258 (11.1)	2315 (100)
Chota	2 (0.3)	76 (12.9)	21 (3.6)	395 (67.2)	94 (16.0)	588 (100)
Macurrungo	36 (2.3)	145 (9.1)	56 (3.5)	1154 (72.1)	209 (13.1)	1600 (100)
Manga-Loforte	0 (0.0)	33 (4.5)	6 (0.8)	613 (82.7)	89 (12.0)	741 (100)
Marrocanhe	1 (1.1)	18 (19.6)	8 (8.7)	64 (69.6)	1 (1.1)	92 (100)
Matadouro	1 (0.9)	62 (54.9)	2 (1.8)	22 (19.5)	26 (23.0)	113 (100)
M-Nhaconjo	84 (2.7)	358 (11.5)	217 (7.0)	1633 (52.6)	813 (26.2)	3105 (100)
Munhava	158 (4.8)	292 (8.9)	134 (4.1)	2288 (70.0)	398 (12.2)	3270 (100)
Nhagau	0 (0.0)	16 (8.0)	1 (0.5)	171 (85.1)	13 (6.5)	201 (100)
P-Militar	0 (0.0)	80 (18.1)	17 (3.9)	301 (68.3)	43 (9.8)	441 (100)
Ponta-gea	142 (4.8)	260 (8.8)	121 (4.1)	2062 (70.1)	358 (12.2)	2943 (100)
Sao-Lucas	5 (1.1)	28 (6.4)	3 (0.7)	362 (83.2)	37 (8.5)	435 (100)
US-Ceramica	0 (0.0)	28 (17.2)	9 (5.5)	95 (58.3)	31 (19.0)	163 (100)
US-M. Mascarenha	31 (2.7)	120 (10.3)	91 (7.8)	852 (73.2)	70 (6.0)	1164 (100)
Beira District	542 (3.1)	1664 (9.4)	772 (4.4)	12,300 (69.7)	2542 (14.4)	17,657 (100)

**Table 2:** First HIV test, by timing and type (PCR, Rapid Test), administered during the first consultations at the 15 ARCCs of the Beira District, 2015–2017.

ARCCs of the Beira District	PCR <8th week		PCR 8th week–9th month		Rapid Test 9th–18th month		Total Tests	
	Test Administered n.	Positive Results n. (%)	Test Administered n.	Positive Results n. (%)	Test Administered n.	Positive Results n. (%)	Test Administered n.	Positive Results n. (%)
Chamba	127	9 (7.1)	153	32 (20.9)	97	26 (26.8)	377	67 (17.8)
Chingussura	1302	39 (2.9)	226	20 (8.8)	565	30 (5.3)	2093	89 (4.25)
Chota	221	13 (5.9)	115	16 (13.9)	324	51 (15.7)	660	80 (12.1)
Macurrungo	821	28 (3.4)	149	19 (12.7)	32	9 (28.1)	1002	56 (5.6)
Manga-Loforte	335	32 (9.5)	267	63 (23.6)	350	24 (6.8)	952	119 (12.5)
Marrocanhe	8	1 (12.5)	36	2 (5.55)	37	6 (16.2)	81	9 (11.1)
Matadouro	8	0 (0.0)	5	1 (20.0)	8	2 (25.0)	21	3 (14.3)
Munhava	1574	101 (6.4)	230	20 (8.7)	1279	96 (7.5)	3083	217 (7.0)
Nhagau	60	5 (8.3)	67	11 (16.4)	127	11 (8.7)	254	27 (10.6)
M-Nhaconjo	1222	63 (5.1)	393	41 (10.4)	1326	144 (10.8)	2941	248 (8.43)
P-Militar	84	10 (11.9)	54	9 (16.6)	199	11 (5.5)	337	30 (8.9)
Ponta-gea	1679	95 (5.6)	227	23 (10.1)	1168	100 (8.6)	3074	218 (7.1)
Sao-Lucas	221	8 (3.6)	87	10 (11.5)	266	9 (3.4)	574	27 (4.7)
US-Ceramica	68	5 (7.3)	13	5 (38.5)	37	4 (10.8)	118	14 (11.9)
US-M. Mascarenha	617	65 (10.5)	194	23 (11.8)	487	38 (7.8)	1298	126 (9.7)
Beira District	8437	474 (5.6)	2216	295 (13.3)	6302	561 (8.9)	16,865	1330 (7.9)

**Table 3:** Type of breastfeeding reported for n. 10,100 HIV Exposed Infants, assisted at the 15 ARCCs of the Beira District, 2015–2017.

ARCCs of the Beira District	Breastfeeding in HIV-Exposed Infants			
	Exclusive n. (%)	Formula n. (%)	Mixed n. (%)	Total n. (%)
Chamba	78 (91.8)	0 (0.0)	7 (8.2)	85 (100)
Chingussura	1179 (89.5)	45 (3.4)	94 (7.1)	1318 (100)
Chota	96 (87.3)	2 (1.8)	12 (10.9)	110 (100)
Macurrungo	872 (92.5)	57 (6.0)	14 (1.5)	943 (100)
Manga-Loforte	260 (86.4)	20 (6.6)	21 (7.0)	301 (100)
Matadouro	33 (94.3)	0 (0.0)	2 (5.7)	35 (100)
Marrocanhe	23 (82.1)	0 (0.0)	5 (17.9)	28 (100)
M-Nhaconjo	1111 (80.1)	208 (15.0)	68 (4.9)	1387 (100)
Munhava	1193 (85.0)	202 (14.4)	9 (0.6)	1404 (100)
Nhagau	146 (84.9)	0 (0.0)	26 (15.1)	172 (100)
P-Militar	126 (69.2)	40 (22.0)	16 (8.8)	182 (100)
Ponta-gea	1288 (86.3)	191 (12.8)	14 (0.9)	1493 (100)
Sao-Lucas	596 (79.7)	118 (15.8)	34 (4.5)	748 (100)
US-Ceramica	49 (59.8)	2 (2.4)	31 (37.8)	82 (100)
US-M. Mascarenha	1355 (74.8)	256 (14.1)	201 (11.1)	1812 (100)
Beira District	8405 (83.2)	1141 (11.3)	554 (5.5)	10,100 (100.0)
